# Efficiency *Eff_syn_* of complex syntheses as multicomponent reactions, its algorithm and calculations based on concrete criteria

**DOI:** 10.3762/bjoc.15.142

**Published:** 2019-06-27

**Authors:** Heiner Eckert

**Affiliations:** 1 Technical University of Munich, Department of Chemistry, Lichtenbergstr. 4, D-85747 Garching, Germany

**Keywords:** efficiency, efficiency algorithm, multicomponent, overall yield, synthesis algorithm, synthesis efficiency, synthesis evaluation

## Abstract

A synthesis efficiency algorithm, which must be based on concrete and reliable criteria, is essential for the evaluation and control of complex chemical synthesis, notably multicomponent reactions (MCRs). An algorithm has been developed to precisely evaluate even highly complex syntheses with regards to their synthesis efficiency *Eff_syn_* as a tool for strict compliance with green chemistry requirements, and for economic progress. The mathematical operations are highly suitable for electronic data processing (EDP). This algorithm is also suitable as a basis for fair cost assessment of complex chemical syntheses.

## Introduction

The ongoing upheavals in the sectors of information technology, energy and electromobility, which are in some cases extremely competitive, mean that ecological and economical aspects of chemistry, as applicable to humans, are increasingly being focused on during this socio-economic transformation. Comprehensive efforts are being undertaken in this field, including in large workshops, e.g., [1].

The efficiency of synthesis forms the core for the evaluation of innovations within synthesis chemistry [2–6] and is the indispensable requirement for a radical simplification of chemical synthesis [3]. Concrete and reliable criteria must be available for this purpose, criteria that can be easily determined and measured, and which can also form the basis for an algorithm. The standard evaluation of a chemical synthesis is traditionally based on the overall yield *y_oa_*. This is the product of all sequential synthesis steps *y_n_* (Equation 1).

[1] $$y_{oa}=\prod_{n=1}^{N}y_{n,\text{ (}n\text{ = 1}\text{, 2}\text{, etc)}}$$

An extreme example for the impact of the overall yield is the tropinone synthesis by Willstätter (*y_oa_* = 0.75%) [7–8] compared to the Robinson–Schöpf synthesis (*y_oa_* = 90%) [9–10]) using a double Mannich reaction, a multicomponent reaction (MCR) [11–13]. The Mannich-3CR is therefore 120 times better than the Willstätter synthesis.

### Criterion overall yield y_oa_

This y_oa_ directly influences the variable costs for the starting and other materials in each synthesis, but not most other (fixed) costs.

### Criterion synthesis step number *n*

Such costs are significant and manifold, deriving from direct costs such as fixed employee and laboratory costs, laboratory rental and maintenance costs, operating costs, i.e., power, water, (gas), inert gas and disposal costs. Standard laboratory activities that are repetitive, such as reactor configuration, filling, reaction monitoring, draining, work-up, preparation of reaction mixture and product isolation, product purification (distillation, recrystallisation, chromatography) and product analysis apply to all synthesis steps. All these costs are similar for each synthesis step *n* and can be said to be constant in the first approximation in cumulo. This provides a second concrete criterion, the synthesis step *n*, which also encompasses and quantifies two factors – “waste prevention” and “energy efficiency” – as requirements for “green chemistry”.

The efficiency of a synthesis, *Eff_syn_* will be defined in Equation 2. The synthesis step *n* in the context of this paper is a practical unit of reactions with supplements that all are run in one pot in one working process without intermediate isolation and purification of the reaction participants. The synthesis step therefore differs somewhat from the normal definition of a reaction step.

[2] $$Eff_{syn}=y_{oa}/N\text{ (}N\text{ = overall number of synthesis steps)}$$

Time influences reactions via their kinetics and is therefore not a primary factor but a soft criterion. This can usually be greatly minimised during cost generation through clever time management of the synthesis planning and can essentially be treated here as a fixed cost.

Table 1 indicates the major impact the synthesis steps *n* have on the efficiency *Eff_syn_* of the synthesis. The range of profitable to useful syntheses decreases drastically with increasing synthesis steps *n*. The detrimental impact of a greater number of steps *n* is shown in the above-mentioned tropinone synthesis by Willstätter (*y_oa_* = 0.75%, *N* = 20, *Eff_syn_* = 0.038% [7–8]), compared with the Robinson–Schöpf synthesis (*y_oa_* = 90%, *N* = 1, *Eff_syn_* = 90% [9–10]). The latter MCR is therefore 2368 times (!) more efficient than the original Willstätter synthesis. Further examples, including the comprehensive synthesis of complex natural substances, can be found in [2–3].

**Table 1:** Overall yields *y_oa_* and synthesis efficiency *Eff_syn_*_._

Overall yields *y_oa_* [%] geometric average yields *y_av_*					Number of steps *n*	Synthesis efficiency *Eff_syn_* [%] geometric average yields *y_av_*				
	
**95**	**90**	**80**	**70**	**60**		**95**	**90**	**80**	**70**	**60**

**95**	**90**	**80**	*70*	*60*	1	**95**	**90**	**80**	*70*	*60*
**90**	**81**	*64*	*49*	*36*	2	**45**	**40**	*32*	*25*	*18*
**86**	**73**	*51*	*34*	*22*	3	*29*	*24*	*17*	*11*	7.3
**81**	*66*	*41*	*24*	13	4	*20*	*17*	*10*	6.0	3.0
**77**	*59*	*33*	17	7.8	5	*15*	*12*	6.6	3.4	1.6
**74**	*53*	*26*	12	4.7	6	*12*	8.8	4.3	2.0	0.78
*70*	*48*	21	8.2	2.8	7	*10*	6.9	3.0	1.2	0.40
*66*	*43*	17	5.8	1.7	8	8.3	5.4	2.1	0.73	0.21
*63*	*39*	13	4.0	1.0	9	7.0	4.3	1.8	4.4	0.11
*60*	*35*	11	2.8	0.6	10	6.0	3.5	1.1	0.28	0.06

These figures may astound some people, but they are the clear results of an impartial analysis. Limitations need to be determined in order to delineate the scope of a meaningful application area.

Ignoring or omitting the number of steps as an essential criterion is a serious issue, for example, if one simply assumes that 4 reactions with 97% yield each are better than a 4CR with 90% yield. The fact that the outlay (fixed costs) during MCR drop by a massive 75% – compared to the 4 separate reactions – is often ignored. And those 4 separate reactions actually have an overall yield of 88.5%. This behaviour is unfortunately very common, culpably inefficient! The overall yield alone does not encompass such facts and any mathematical treatment needs to bring together all primary criteria.

MCRs have a high material and energy efficiency, and their atom balance is quite outstanding. Product purification is usually simple. All this reduces waste to a minimum. Due to the very weak negative reaction enthalpies, MCRs are also usually safe processes. The shortening of the synthesis through drastic reduction of the number of steps *n* leads to a strong waste prevention, which can be quantitatively measured through the synthesis efficiency in Equation 2 (Table 1).

## Results and Discussion

In practice, there are problems with complete calculations of overall yields for complex syntheses, particularly when several precursors (2 or more) need to be included in the calculation, which is almost always the case with MCRs. All such reactions are parallel reactions and do not have any sequential character with respect to each other, instead they are cumulative, whereby the parallel reaction groups have different numbers of individual reactions *n*. The yields *y_n_* then need to be weighted with these *m* values and the arithmetic mean *y_am_* calculated as shown in Equation 3.

[3] $$y_{am}=\sum_{j=1}^{J}(y_{j}*m_{j})/\sum_{j=1}^{J}m_{j}$$

### Algorithm

If you insert Equation 1 in Equation 3, and then insert this in Equation 2, you will obtain the efficiency algorithm Equation 4. This has already been described in a general manner in [2–3]. The weighting of the parallel reactions results in the precise value y_oa_.

[4] $$y_{oa}=\prod_{k=1}^{K}\left[\frac{\sum_{j=1}^{J}\left(\prod_{n=1}^{N}y_{n}*m_{j}\right)}{\sum_{j=1}^{J}m_{j}}\right]{}_{}^{}\begin{array}{ll} K= & \text{number of main reaction steps}\\ J= & \text{number of branches}\\ N= & \text{number of synthesis steps}\\ m= & \text{number of weightings}\\ y & \text{weighted yields} \end{array}$$

The algorithm is broad in scope and can be used in many ways as required through the introduction of constants in the variables *c*_1_, *c*_2_ and invariable C. This permits the inclusion of soft criteria, such as suitable resources, time (see discussion above) and process control, in the cost analysis of complex syntheses (Equation 5).

[5] $$\text{Synthesis costs = }c_{1}*N/c_{2}*y_{oa}+C$$

An App based on this algorithm can offer an effective way to obtain a rapid overview of the total or partial synthesis. It can be used to evaluate and even control the synthesis from various aspects, including how it is affected by soft criteria.

### Case study

**Scheme 1:**
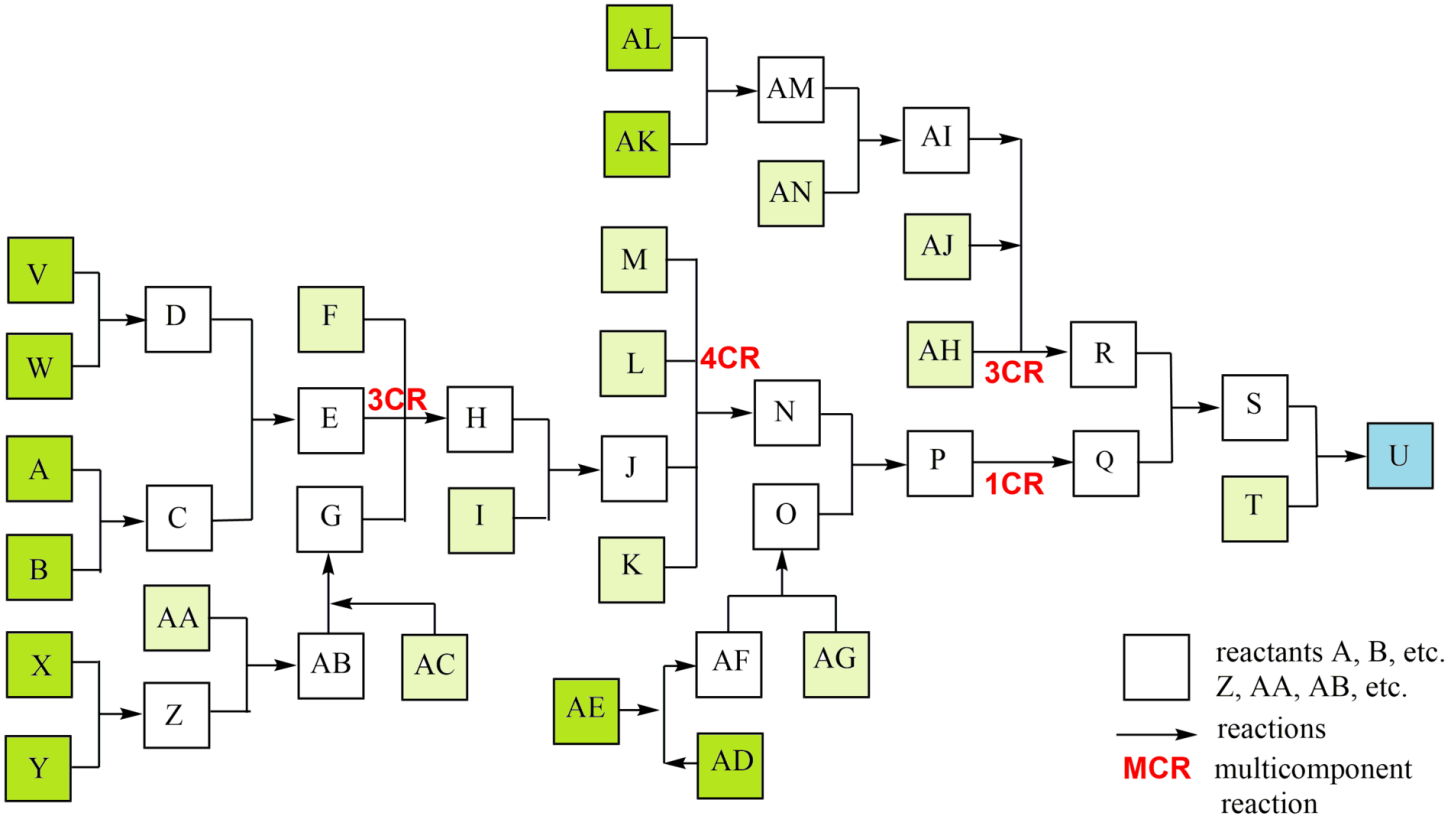
Case study of a complex synthesis, parallel reactions. 10 possible synthesis STMs in green, other 12 STMs in light green, TM U in blue.

All listed and possible constellations of reactions and reaction groups in a complex synthesis are shown in a flow diagram (Scheme 1) and are presented in a detailed case study; the data were inserted into the general efficiency algorithm [2–3]. Although publications usually only show the synthesis path with the most spectacular molecules, such as the target molecule (TM), the total synthesis with all reactions is essential for production. The quantity of potential start molecule (STM) sets also rises strongly in complex syntheses. The example shows 5 STM sets (consisting of 10 STMs) with which the synthesis can be started, as well as 12 other STMs.

**Scheme 2:**
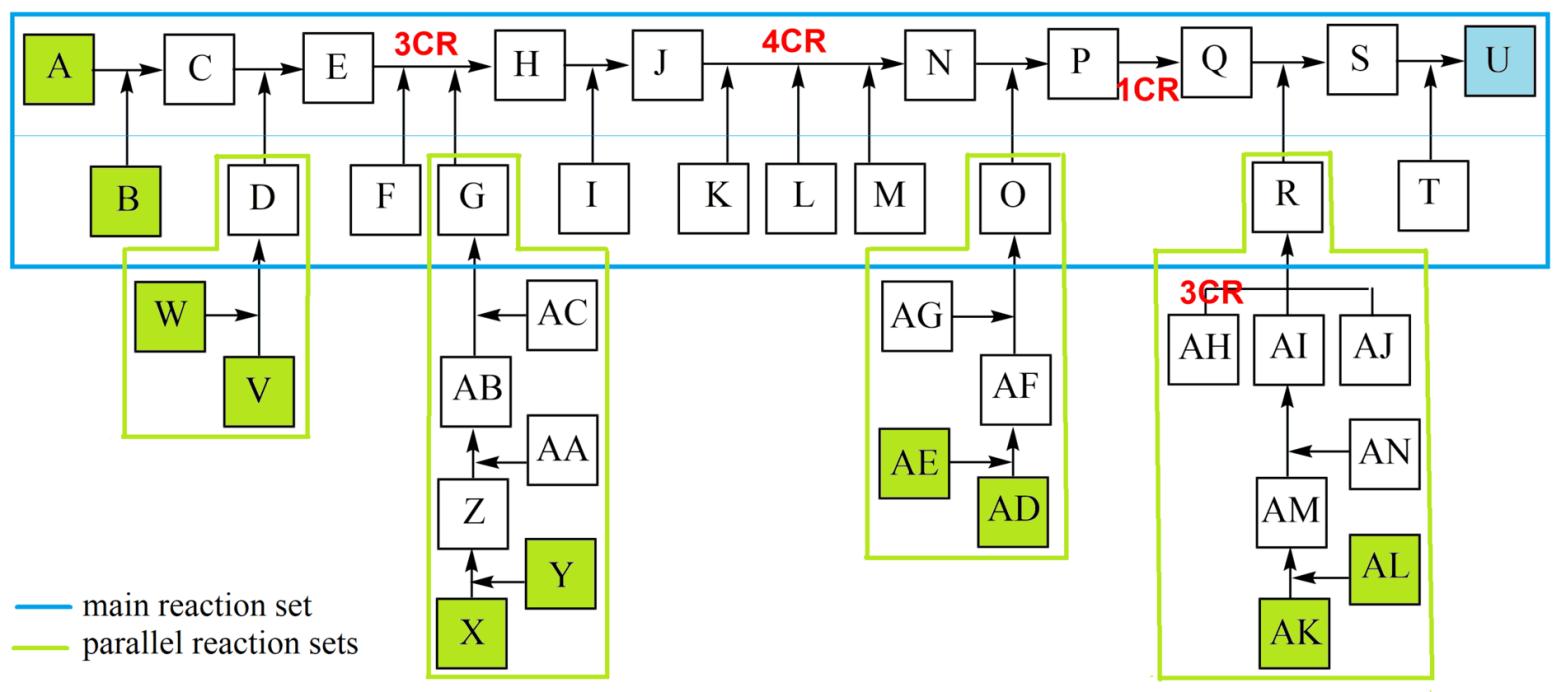
Ordered reactions of the above complex synthesis to a main reaction set and connected parallel reaction sets.

To have a better overview in this study (Scheme 1), reactions are ordered to a main reaction set including two MCRs (reactants A-T, TM U), connected with 4 parallel reaction sets including a 3CR (Scheme 2).

### Overall yield *y_oa_* and efficiency *Eff_syn_* calculation of J in case study

**Scheme 3:**
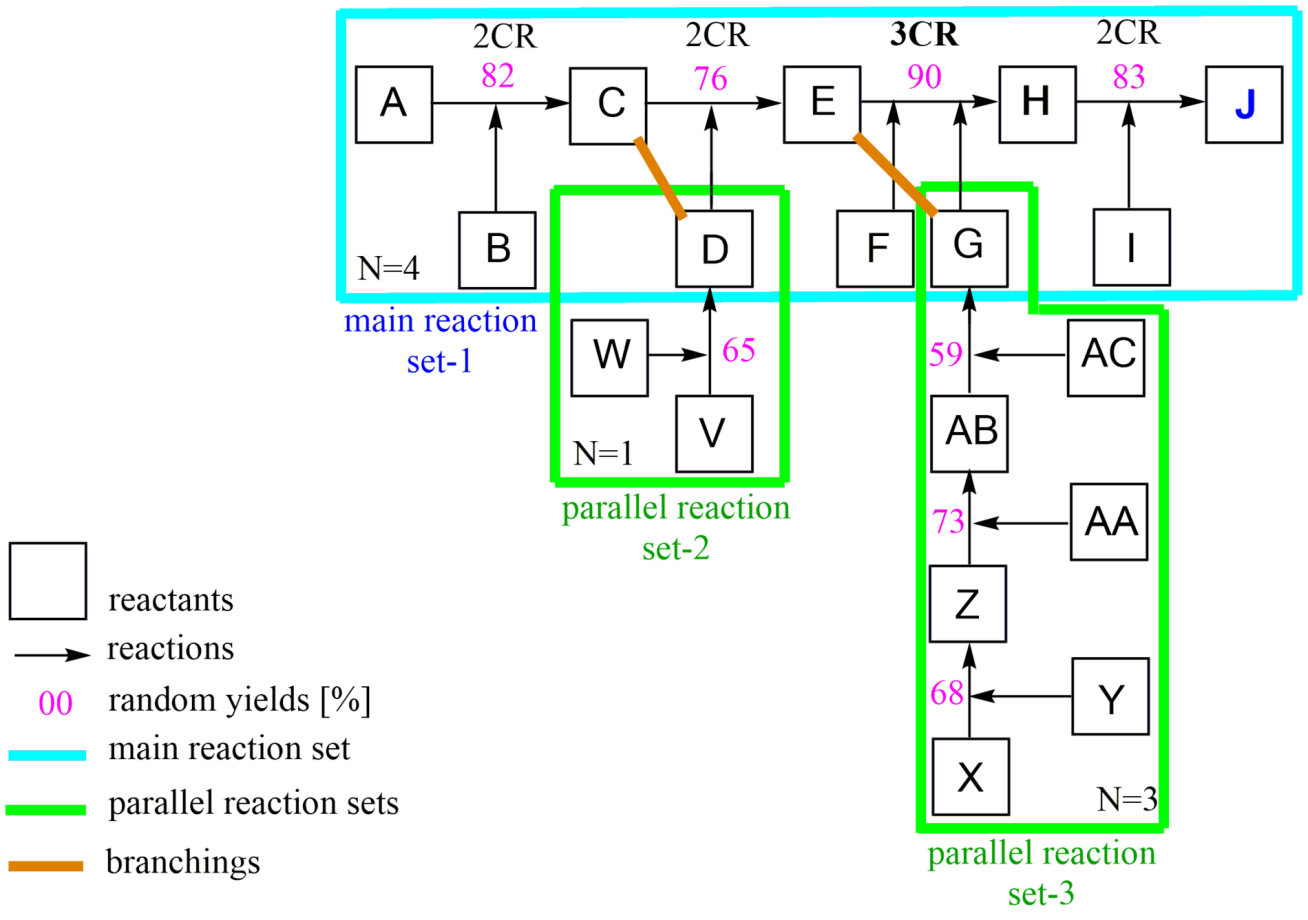
Section A–J case study of Scheme 2 with operations of y_oa_ calculation.

As a practical exercise, random numbers were added to the part A–J with both parallel reactions V–D and X–G in Scheme 3, and the overall yield *y_oa_* was then calculated incrementally with the algorithm in Equation 4. Expediently, a main reaction (set-1) to which the parallel reactions are linked (set-2, set-3) is set up. The weighted arithmetical mean of the yields for each reaction set is formed at the connection forms. The overall yield *y_oa_* of the total synthesis is determined through the sequential operation of the main reaction sets. The latter can be determined using the following calculation method.

### Calculation method

Split the main reaction set-1 at the connection points with the parallel reactions into set-1, set-2, set-3, then use Σ(Π*y_n_*)*_j_* to calculate the weighted mean yield values for the two branches C, D and E, G. The latter values, together with the values from Π[Σ(Π*y_n_*)*_j_*]*_k_*, deliver the overall yield *y_oa_* of the total synthesis according to Equations 6–8.

Parallel reactions set-2 to main reaction position (C), operation Σ(Π*y_n_*)*_j_*

[6] $$\begin{array}{c} y(C,D)=1/2*[y(A-C)*1+y(V-D)*1]\\ =1/2*[0.82*1+0.65*1]\\ =1/2*1.47=0.735 \end{array}$$

Parallel reactions set-3 to main reaction position (E), operation Σ(Π*y_n_*)*_j_*

[7] $$\begin{array}{c} y(E,G)=1/4*[y(C-E)*1+y(X-G)*3]\\ =1/4*[0.76*1+(0.68*0.73*0.59)*3]\\ =1/4*[0.76+0.87]=0.41 \end{array}$$

Sequential main reactions set-1, operation Π[Σ(Π*y_n_*)*_j_*]*_k_*

[8] $$\begin{array}{c} y(A-J)=y[C,D)*(E,G)*(E-J)]\\ =0.735*0.41*0.90*0.83\\ y_{oa}=0.23\;(22.5\%) \end{array}$$

This synthesis consists of *n* = 8 synthesis steps, so the synthesis efficiency is


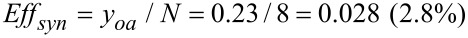


### Modification of the calculation execution

Most chemists look exclusively at the interesting target molecule (TM) of a synthesis and only follow that path from STM to TM, while blanking out everything else. The second method for calculating *y_oa_* in complex syntheses is probably easier in this case. Here, the yield y(A–J) of the sequential main reaction set-1 is calculated (Equation 9) and added to the branches with the parallel reaction modification factors *mf*. This is equivalent to the quotients from the dividends C, D (from Equation 6) or E, G (from Equation 7) and the divisors A–C or E–G (Equations 10 and 11). The result from Equation 9 is multiplied with both modification factors mf to obtain the overall yield *y_oa_* (Equation 10).

[9] y(A−J)=0.82∗0.76∗0.9∗0.83=0.466

[11] mf(C,D)=0.735/0.82=0.896

[12] mf(E,G)=0.41/0.76=0.539

[10] $$\begin{array}{l} y_{oa}=y(A-J)*mf(C,D)*mf(E,G)\\ y_{oa}=0.466*0.896*0.539=0.23\;(22.5\%) \end{array}$$

Some useful strategic and practical applications demonstrate the enormous influence of branching on the overall yield *y_oa_* and synthesis efficiency *Eff_syn_*.

### Fragment strategy: fragment linking in peptides synthesis

**Scheme 4:**
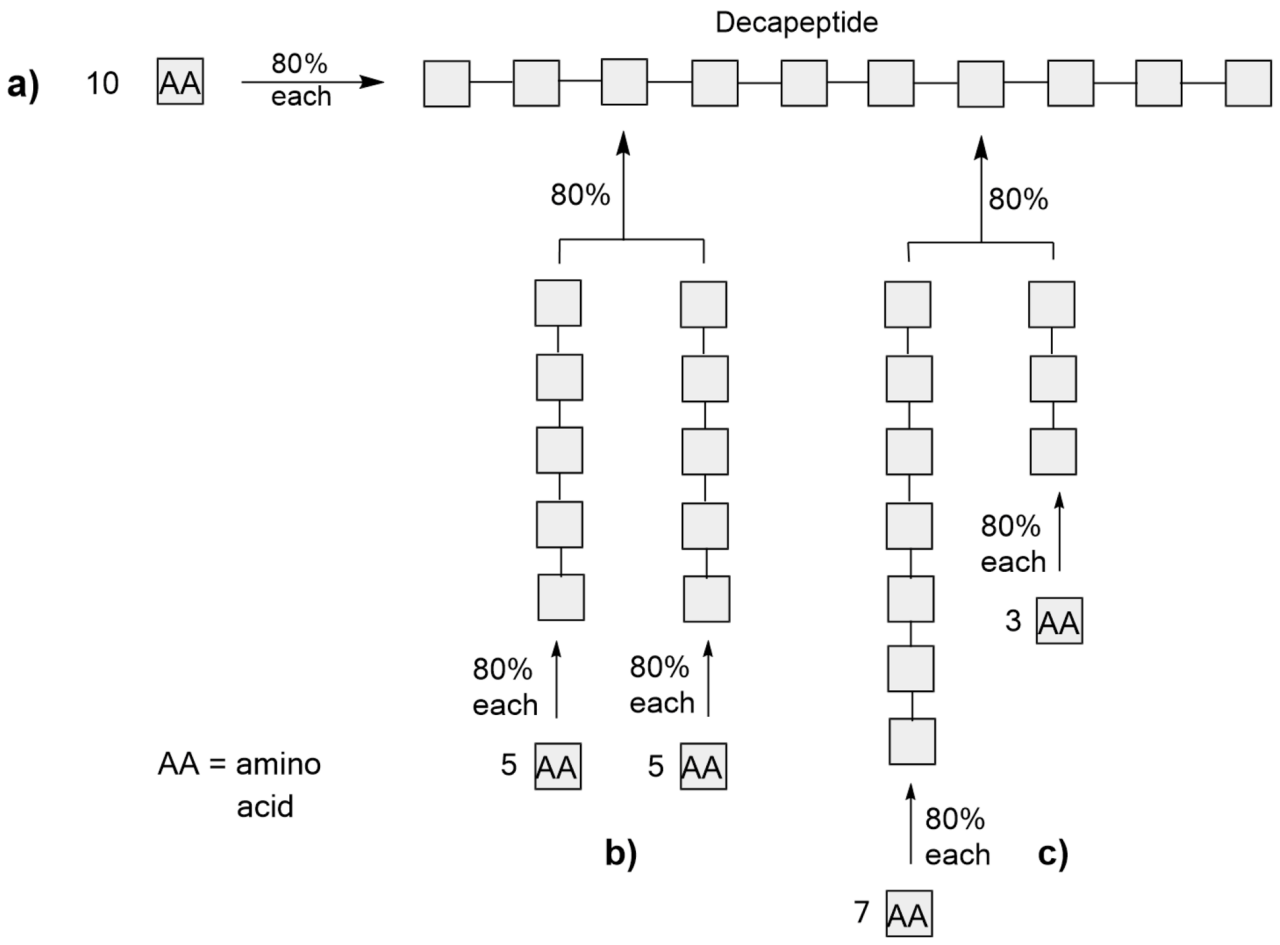
Sequential synthesis and fragment linking of a decapeptide with comparison of results in Equations 13–15.

In synthetic peptide chemistry, amino acids are sequentially built up to form long oligo/polypeptides. For reasons of transparency, we have assumed the same yield of 80% in each step during the synthesis of a decapeptide in order to clearly indicate the effect of the branching (Scheme 4).

Three cases are discussed here:

1. sequential linking of the 10 amino acids (Scheme 4a);
2. sequential synthesis of two pentapeptides and the subsequent linking to form a linear decapeptide (Scheme 4b);
3. sequential synthesis of two non-identical peptides (1 heptapeptide and 1 tripeptide) and their linking to form a linear decapeptide (Scheme 4c);

### Calculation by means of the algorithm

[13] $$\text{a) }y_{oa}=0.8^{9}=0.13\;(13\%)$$

[14] $$\text{b) }y_{oa}=[(0.8^{4}+0.8^{4})/2]*0.8=0.41*0.8=0.33\;(33\%)$$

[15] $$\begin{array}{c} \text{c) }y_{oa}=[(0.8^{6}*6+0.8^{2}*2)/8]*0.8\\ =[(0.26*6+0.64*2)/8]*0.8\\ =[(1.56+1.28)/8]*0.8\\ =0.355*0.8=0.284\;(28.4\%) \end{array}$$

The results are impressive. A up to 2.5-fold yield can be achieved depending on the configuration of the fragment linking, and the algorithm delivers rapid results. The number of steps is, however, only conditionally reduced (with identical steps) and this must be taken into consideration when calculating the synthesis efficiency *Eff_syn_*. The fragment strategy can of course be applied without limitations to other syntheses of this type.

### MCR Strategy: Ugi-4CR in ecteinascidin-743 total synthesis

As described above, yields can be significantly increased by using the fragment strategy. However, the fragment strategy is limited to 2 components, while an MCR provides multiple components for linking, and also generates its own structure which is capable of extensions like the domino reaction types [5].

**Scheme 5:**
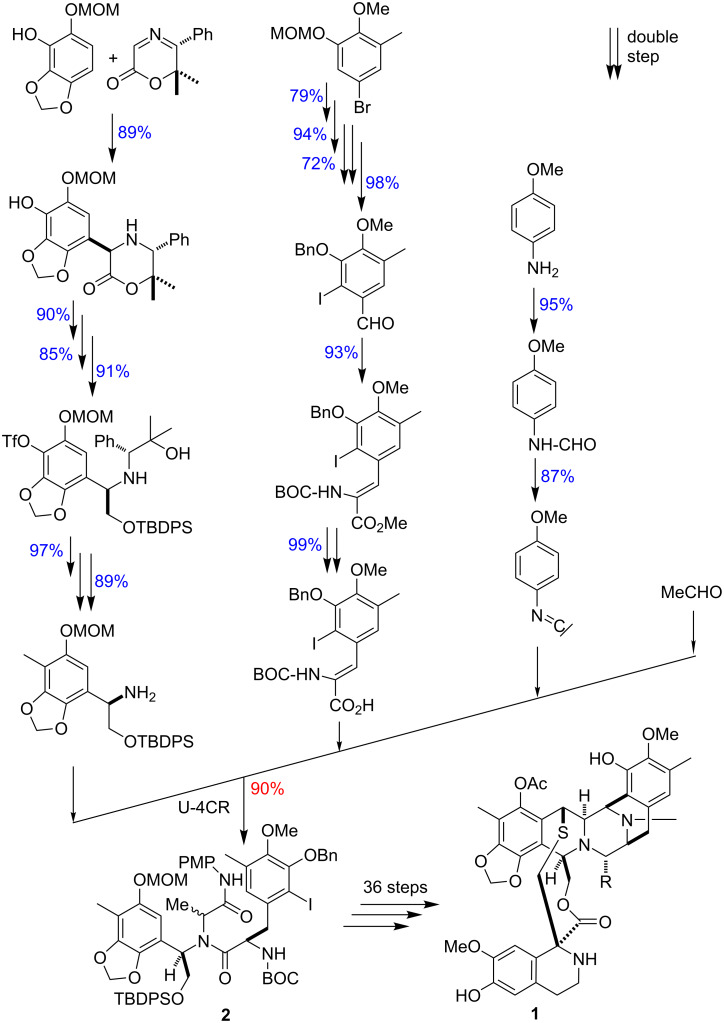
U-4CR with 17 precursors in the total synthesis of Et-743 (**1**) [14].

An Ugi reaction is used in the total synthesis of the extremely potent antitumor agent ecteinascidin-743 (Et-743, **1**) by Fukuyama to form a large part of the skeleton **2** (Scheme 5). All data for the U-4CR are available in the literature [14]. The precursors of the Ugi reaction consist of 3 reaction chains of 6, 6 and 2 links, respectively.

### Calculation by means of the algorithm

The calculation of the overall yield *y_oa_* of **2** is based on the data basis for the total synthesis of ecteinascidin-743 found in the literature [14], and the data for 4-methoxyphenylisocyanide is provided from the author, referred to Equations 16–20.

[16] 0.89∗0.9∗0.85∗0.91∗0.97∗0.89=0.535

[17] 0.79∗0.94∗0.72∗0.98∗0.93∗0.99 = 0.482

[18] 0.95∗0.87=0.827

[19] (0.535∗6+0.482∗6+0.827∗2)/14=7.76/14=0.554

[20] $$y_{oa}=\;0.554*0.9=0.499\;(50\%)$$

An outstanding result is shown for U-4CR, including the *N* = 14 (from real 17; three are double steps) precursors forming **2** with 50% overall yield. Synthesis efficiency is *Eff_syn_* = 2.9%, due to the high step number of 17 (real number of precursors). The same synthesis in linear architecture does not exist. A fictive comparison with the same dataset in a completely sequential reaction sequence results in a fictive yield of **2** *y_oa_*(fictive) = 0.192 (19%). The significant difference is due to the MCR itself and primarily the linked parallel reactions of the 3 precursors, as can be clearly seen in Scheme 5. These results favour the use of the MCR strategy with the Ugi reaction and provide an increase in yield by 2.6 times that of a linear solution.

### MCR strategy: novel MCR as key step in total synthesis of (+)-20*S*-camptothecin

**Scheme 6:**
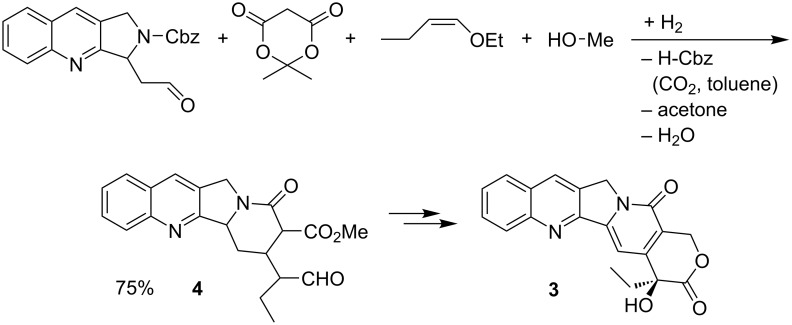
Synthesis of **4** as key step of (+)-20*S*-camptothecin (**3**) total synthesis.

Another typical example for the simplification of a complex chemical synthesis [2–3] is the total synthesis of the extremely potent antitumor agent (+)-20*S*-camptothecin (**3**), which has been a highly effective agent for decades now. This total synthesis by Tietze uses a 4CR, specifically generated for this reaction from an aldehyde, meldrum’s acid, enol ether and methanol, as a key step in the synthesis to **4** (Scheme 6) [15]. This step-saving strategy for generating novel MCRs is a fast track towards the ubiquitous use of MCRs in complex syntheses. This algorithm (Equation 4) is an essential tool for the rational evaluation and synthesis control of MCRs.

## Conclusion

The general efficiency algorithm [2–3], and the calculation methods (Equations 3–20) developed from that algorithm, can be used to precisely evaluate even highly complex syntheses and quantitatively compare them with alternative syntheses with regards to their synthesis efficiency *Eff_syn_*. The mathematical operations are highly suitable for electronic data processing (EDP), as is the algorithm itself. Due to the concrete criteria, this algorithm is also suitable as a basis for fair cost assessment of complex chemical syntheses. Fragment linking reactions, and an ecteinascidin-743 total syntheses including an Ugi reaction, as well as a 20*S*-camptothecin total synthesis based on a 4CR specifically generated for this purpose, were discussed. This demonstrates the high efficiency of the MCR application and its intrinsic suitability for green chemistry.

## References

[R1] 1http://low-carbon-urban-development-germany-china.org/wp-content/uploads/2016/04/Weber-Sites-Consulting-Energy-Efficient-Technologies-in-Chemical-Industry-DE.pdf

[2] Eckert H (2017). Molecules.

[3] Eckert H (2017). Radical and Concerted Simplification of Chemical Synthesis.

[4] Eckert H Radical simplification of chemical synthesis by MCRs Strategies. Book of abstract, MCR-2018.

[5] Tietze L F (2014). Domino Reactions: Concepts for Efficient Organic Synthesis.

[6] Bienaymé H, Hulme C, Oddon G, Schmitt P (2000). Chem – Eur J.

[7] Willstätter R (1901). Justus Liebigs Ann Chem.

[8] Smit W A, Bochkov A F, Caple R (1998). Organic Synthesis: The Science behind the Art.

[9] Robinson R (1917). J Chem Soc, Trans.

[10] Schöpf C (1937). Angew Chem.

[11] Zhu J, Bienaymé H (2005). Multicomponent Reactions.

[12] Mueller T J J (2014). Multicomponent Reactions.

[13] Huang Y, Yazbak A, Dömling A, Zhang W, Cue B C Multicomponent reactions. Green Techniques for Organic Synthesis and Medicinal Chemistry.

[14] Endo A, Yanagisawa A, Abe M, Tohma S, Kan T, Fukuyama T (2002). J Am Chem Soc.

[15] Tietze L F, Bischoff M, Khan T A, Liu D (2017). Chem Heterocycl Compd.

